# Effect on cardiovascular disease risk factors of interventions to alter consultations between practitioners and patients with type 2 diabetes: A systematic review and meta‐analysis of trials in primary care

**DOI:** 10.1111/hex.12546

**Published:** 2017-02-28

**Authors:** Hajira Dambha‐Miller, Andrew J. M. Cooper, Ann Louise Kinmonth, Simon J. Griffin

**Affiliations:** ^1^ Primary Care Unit Department of Public Health and Primary Care University of Cambridge Cambridge UK; ^2^ MRC Epidemiology Unit Institute of Metabolic Science University of Cambridge Cambridge UK

**Keywords:** cardiovascular disease, consultation, doctor‐patient relationship, patient‐practitioner interactions, type 2 diabetes

## Abstract

**Objective:**

To examine the effect on cardiovascular (CVD) risk factors of interventions to alter consultations between practitioners and patients with type 2 diabetes.

**Search Strategy:**

Electronic and manual citation searching to identify relevant randomized controlled trials (RCTs).

**Inclusion Criteria:**

RCTs that compared usual care to interventions to alter consultations between practitioners and patients. The population was adults aged over 18 years with type 2 diabetes. Trials were set in primary care.

**Data extraction and synthesis:**

We recorded if explicit theory‐based interventions were used, how consultations were measured to determine whether interventions had an effect on these and calculated weighted mean differences for CVD risk factors including glycated haemoglobin (HbA_1c_), systolic blood pressure (SBP), diastolic blood pressure (DBP), total cholesterol (TC), LDL cholesterol (LDL‐C) and HDL cholesterol (HDL‐C).

**Results:**

We included seven RCTs with a total of 2277 patients with type 2 diabetes. A range of measures of the consultation was reported, and underlying theory to explain intervention processes was generally undeveloped and poorly applied. There were no overall effects on CVD risk factors; however, trials were heterogeneous. Subgroup analysis suggested some benefit among studies in which interventions demonstrated impact on consultations; statistically significant reductions in HbA_1c_ levels (weighted mean difference, −0.53%; 95% CI: [−0.77, −0.28]; *P*<.0001; *I* ^2^=46%).

**Conclusions:**

Evidence of effect on CVD risk factors from interventions to alter consultations between practitioners and patients with type 2 diabetes was heterogeneous and inconclusive. This could be explained by variable impact of interventions on consultations. More research is required that includes robust measures of the consultations and better development of theory to elucidate mechanisms.

## 1 INTRODUCTION

The processes by which patients and their practitioners interact during consultations are a potentially important modifiable context for optimizing the delivery of health care. National Health Service (NHS) policy, national guidelines and medical training emphasize the value of consultations in improving health‐care quality and outcomes.^1^, ^2^ Observational studies suggest a potential therapeutic effect of consultations in type 2 diabetes; better patient‐reported experiences of consultations have been associated with lower cardiovascular (CVD) risk factors, including glycosylated haemoglobin (HbA_1c_), blood pressure and lipids.^3^, ^4^, ^5^, ^6^, ^7^, ^8^ While the observational evidence is consistent, evidence from trials of interventions to alter consultations remains inconclusive. This may be due to variations in context, potential confounding variables in observational studies or reverse causality. Alternatively, this may be due to suboptimal intervention development or delivery such that impact on consultations is limited. The many sources of heterogeneity identified in previous reviews among patient groups, intervention definition, health‐care context or outcomes may also contribute to inconsistency in trial findings. This heterogeneity has not previously been thoroughly examined and explained, thus limiting scientific value and clinical application.^8^, ^9^

A review by our group in 2004 drew attention to these difficulties and also to other methodological limitations including the lack of description of mechanisms or measurement of variables to test these.^8^ However, our previous review like others combined primary and secondary care and merged multiple chronic diseases.^8^, ^9^, ^10^ Only one previous review has examined randomized controlled trials (RCTs) of interventions to alter consultations between patient and practitioners, specifically among people with type 2 diabetes. This was published over 10 years ago, did not include a meta‐analysis and was not set in primary care.^5^ The findings from these previous reviews are therefore not directly generalizable to diabetes patients in primary care today, where the vast majority of type 2 diabetes is now managed in the UK. Since our last review, additional RCTs have been conducted. Accordingly, we undertook a systemic review and meta‐analysis of RCTs specifically set in primary care and including patients with type 2 diabetes. The primary aim was a quantitative synthesis of the literature to examine the effect of interventions to alter consultations between practitioners and patients with type 2 diabetes on CVD risk factors including glycosylated haemoglobin(HbA_1c_), systolic blood pressure(SBP), diastolic blood pressure(DBP), total cholesterol (TC), LDL cholesterol (LDL‐C) and HDL cholesterol (HDL‐C). We also examined heterogeneity and explored potential sources to explain this.

## 2 METHODS

### 2.1 Search strategy

We performed an electronic search of the Cochrane Central Register of Controlled Trials (CENTRAL), MEDLINE and EMBASE from inception to August 2014. Monthly automated electronic search updates were then set up and the results screened until June 2016. Manual searching was also conducted from the reference lists of included studies, previous reviews on similar topics and conference abstracts.^8^, ^9^, ^11^ Search terms were identified from earlier reviews related to consultations between patient and practitioner (full search strategy available from the authors on request).^12^, ^13^ We used both free text and MESH terms and did not apply language or publication restrictions.

### 2.2 Selection criteria

**Figure 1:**
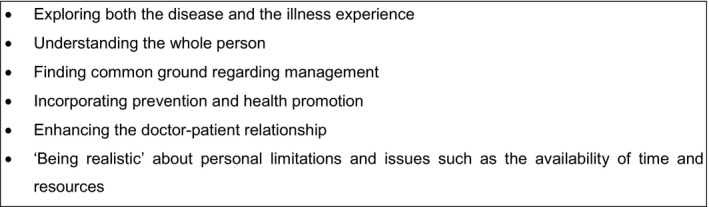
**Conceptual framework to define the consultation** 12

We included RCTs that compared usual care to interventions to alter consultations between practitioners and patients. The population was adults aged over 18 years with type 2 diabetes. Trials were set in primary care, or in community clinics that were comparable to UK primary care. Interventions to alter consultations are diverse and we therefore used a theoretical framework based on the Stewart et al. model of the patient‐centred consultation for the purposes of our review, as shown in Figure 1.^8^, ^12^ This is the most highly cited definition of the consultation in UK primary care. We included interventions that targeted at least one component of the consultation according to this model.^14^ Included trials reported data on at least one of the following outcomes: HbA_1c_, SBP, DBP, total cholesterol, LDL‐C or HDL‐C levels.

### 2.3 Process indicators

We examined descriptions of interventions and recorded if these were based on any explicit theoretical rationale or framework. We also recorded whether trials included any measures of the consultation to demonstrate an effect of the intervention on the consultation.

### 2.4 Data extraction and validity assessment

One author (HDM) screened all titles, and a second author (AC) independently screened 10% of titles. Both authors then independently reviewed all included abstracts and full text papers for data extraction and methodological quality. Quality was appraised by examining each paper's description and making a judgement regarding potential bias using the criteria of randomization, sequence generation allocation concealment, blinding, comparability of groups at baseline, incomplete data reporting and selective reporting.^15^ For each criterion, we recorded potential risk of bias according to three categories: yes, no or risk unclear. Disagreements were documented and resolved by discussion. We also met with a third reviewer three times (SJG), and a fourth reviewer (ALK) was consulted on one occasion as well. Our main discussions related to the fact that data were not always reported as mean difference and 95% CIs. There were also some discussions about one trial that was not set in primary care and was instead based in a community outreach clinic. As a result of these discussions, we included a sensitivity analysis with and without this trial which is described below. We tested for evidence of publication bias with a standard funnel plot generated using the RevMan software.

### 2.5 Data synthesis and analysis

We pooled weighted mean difference and 95% CIs in a random‐effects model that was then inserted into the RevMan software for statistical analysis.^15^, ^16^ We also sought the advice of two statisticians during the data extraction phase. If trials reported on the outcome of interest but the data were not in a form that could permit meta‐analysis, we requested additional data from authors. For example, trials reported the median with a range, instead of mean and standard deviation. Data were requested from five authors. Rather than omitting the trials from the meta‐analysis if no author response was received, which could introduce bias and reduce statistical power, we used established imputation methods with the advice of statisticians.^14^, ^17^ Data reported from the same trial at different time points were included only once in the analysis. We used the *I* ^2^ statistic to assess heterogeneity for each outcome. *I* ^2^ values of 25%, 50% and 75% represented low, medium and high degrees of heterogeneity, respectively.^15^ Where high levels of heterogeneity were detected, we conducted subgroup analysis to understand the reasons for this heterogeneity including assessment of the following on the effect on CVD risk factors: (i) whether trials reported consultation measures that suggested the interventions had an effect on altering the consultation, (ii) trial duration, (iii) whether the intervention targeted the patient, practitioner or both, (iv) whether the practitioners were doctors, nurses, allied health professionals or a combination, (v) diabetes severity based on HbA_1c_ level and (vi) whether trials were set outside of the UK. Further, we conducted a sensitivity analysis with the meta‐analyses recalculated after excluding a trial that was not set in primary care but instead in a clinic that was comparable to UK primary care.

## 3 RESULTS

**Figure 2:**
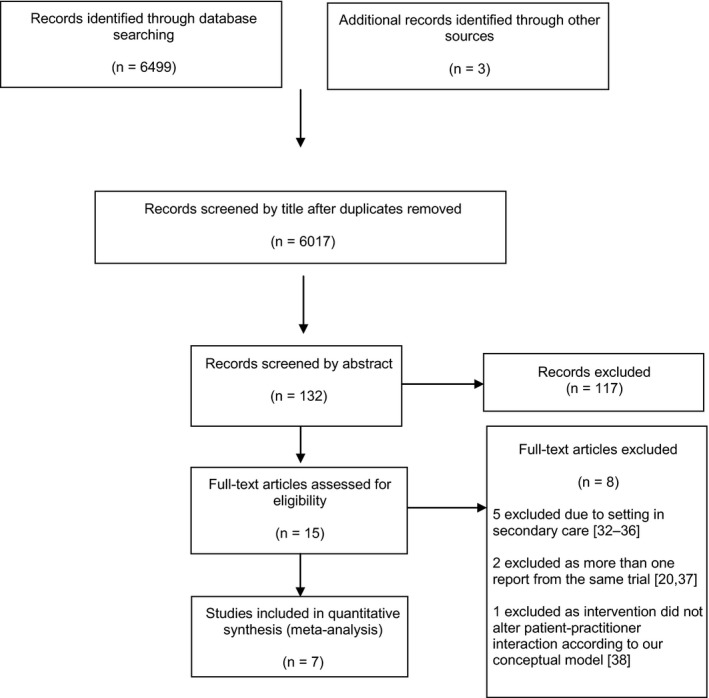
The flow of information through the systematic review

We identified 6502 potentially relevant studies. Of these, 485 were removed electronically as they were either duplicates or clearly fell outside our inclusion criteria, and we screened the remainder by title. In the second round, 132 studies remained. Following screening of abstracts, we identified 15 studies for a third round of screening with full text assessment. Seven RCTs met the inclusion criteria. The flow of information through the systematic review is demonstrated in Figure 2.

### 3.1 Study characteristics

The characteristics of the seven included RCTs are summarized in Table 1. Three trials were conducted in the USA, three in the UK and one in Denmark. The majority were set in primary care with the exception of one study, which was in a general internal medicine clinic.^18^ However, the trial adopted a chronic care model that is comparable to the UK primary care system, and after discussions between reviewers, we agreed to include it.^19^ Trial duration ranged from 12 months to 6 years, and the sample sizes ranged from 83 to 875 participants of which 47%‐77% were male. In total, this review included 2277 participants with varying levels of glycaemic control; mean baseline HbA_1c_ values across studies ranged from 6.8% to 11.6% in the six trials that reported this information. Participant characteristics are summarized in Table 2.

**Table 1:** Characteristics of included studies

First author	Year	Country	Intervention	Duration	Number of participants	Risk of bias	Lost to follow‐up
Christian^31^	2008	USA	Patient self‐management and goal setting on health behaviours through computer program. Accompanying computer report is also produced for the practitioner to encourage personalized and patient‐specific counselling interactions. Practitioner additionally received 3 hours of training on brief motivational interviewing to encourage behavioural change during interactions	12 months	310	Blinding Y Sequence generation N Allocation Concealment N Comparability of groups at baseline N Incomplete data reporting N Selective reporting N	12%
Deakin^20^	2006	UK	Structured diabetes education programme for patients, based on theories of patient empowerment and discovery learning which encourages skills confidence and self‐management	14 months	341	Blinding Y Sequence generation N Allocation Concealment N Comparability of groups at baseline N Incomplete data reporting N Selective reporting N	7%
De fine Olivarius^22^	2013	Denmark	Regular follow‐up of patients with individualized goal setting supported by prompting of practitioners, clinical guidelines, feedback, and continuing medical education support	6 years	874	Blinding Y Sequence generation N Allocation Concealment N Comparability of groups at baseline Y Incomplete data reporting N Selective reporting N	9%
Kinmonth^23^	1998	UK	Practitioner training days on the evidence and skills of patient‐centred care. Information booklet provided as well. Patients also provided with information leaflet to encourage participation	12 months	360	Blinding Y Sequence generation N Allocation Concealment N Comparability of groups at baseline UNCLEAR RISK Incomplete data reporting N Selective reporting N	15%
Piatt^13^	2010	USA	Intervention A: Collaborative care model involved both patient education through diabetes self‐management programme. Practitioner education given through problem based learning session. Community system redesign for diabetes services developed. Certified Diabetes educator placed in the practices for extra support Intervention B: Involves practitioner only education through one problem based learning session and as required access to certified Diabetes educator who is accessible over the course of 6 months, but not placed in practice	36 months	119	Blinding Y Sequence generation UNCLEAR RISK Allocation Concealment N Comparability of groups at baseline N Incomplete data reporting N Selective reporting N	52%
Pill^21^	1998	UK	Training sessions for practitioners on patient‐centred care followed by continuing educational support group meetings and written material	36 months	190	Blinding Y Sequence generation N Allocation Concealment N Comparability of groups at baseline N Incomplete data reporting N Selective reporting N	28%
Ralston^18^	2009	USA	Patient activation and engagement through web‐based program. The program allows the patients access to electronic medical records, email communication with practitioners, feedback on blood glucose, educational material and interactive online diary for entering information about exercise, diet and medication	12 months	83	Blinding Y Sequence generation N Allocation Concealment N Comparability of groups at baseline N Incomplete data reporting N Selective reporting N	11%

N, No risk of bias present; Y, Yes risk of bias present.

**Table 2:** Characteristics of study participants

First author	Age (years)	Sex (% male)	Ethnicity (% non‐white)	Baseline HbA_1c_ (%)
Christian^31^	53	47	50	8.2
Deakin^20^	61	52	Not reported	7.7
De fine Olivarius^22^	65	52^a^	Not reported	10.2^a^
Kinmonth^23^	57	62	Not reported	Not reported
Piatt^13^	65	48	10	6.8
Pill^21^	58	64	Not reported	11.6
Ralston^18^	41	77	81	8.1

Age and HbA_1c_ reported as means unless stated.

a Indicates data reported as medians.

All RCTs targeted more than one aspect of the consultation according to our theoretical model as shown in Table 3. There were a total of eight interventions across the seven trials. Interventions were frequently complex behavioural interventions aimed at achieving behavioural change although we found that no specific frameworks had been used to underpin the interventions or designs. Four of the included RCTs did not include any theoretical rationale to explain how interventions might have an effect on outcomes, and no trials directly related these to testing of potential underlying mechanisms.

**Table 3:** Characteristics of the intervention according to the Stewart et al. consultation model 12

First author	Exploring both the disease and the illness experience	Understanding the whole person	Finding common ground regarding management	Incorporating prevention and health promotion	Enhancing the doctor‐patient relationship	“Being realistic” about personal limitations and issues such as the availability of time and resources
Christian^31^	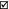	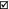	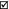	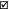	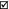	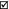
Deakin^20^		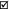	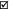	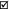		
De fine Olivarius^22^	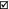	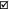	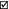	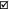	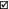	
Kinmonth^23^	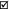	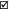	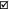	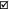	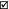	
Piatt A^13^	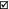	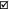	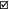	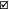	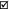	
Piatt B^13^	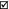	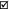	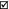		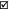	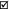
Pill^21^	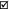	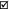	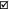	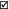	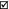	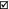
Ralston^18^	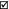	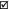	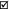	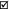		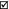

Piatt study had two different intervention arms and is therefore identified as “A” and “B”.

Interventions to alter consultations were expectedly diverse, and most of these were not conducted within face‐to‐face consultations between patients and practitioners. Instead of altering elements of the traditional face‐to‐face consultation model, interventions seemed to run parallel to these in order to augment the consultation. This was particularly observed in more recent trials that included type 2 diabetes patients working with case managers,^18^ dieticians^20^ or diabetes educators,^13^ as well as group education sessions^20^ or sharing medical records with patients through web‐based care.^18^ In the newer studies, we observed a greater move towards multidisciplinarity and multisystem redesign aiming to enable patients and empower them to alter the consultation with the practitioner.

Across trials, quality varied with the biggest source of bias related to allocation concealment; however, interventions cannot be fully concealed from practitioners and patients in these sorts of studies. A summary of risk of bias assessment is included. (Table 1)

### 3.2 Process indicators

Only one trial included an independent objective measure of the consultation through the analysis of audio‐recordings of consultations.^21^ Five trials included self‐report measures of the consultation including provider feedback^22^; patient‐completed questionnaires on satisfaction, patient empowerment and patient diabetes knowledge^20^; and consultation communication scores.^23^ One trial observed the number of times that patients had accessed their medical records, uploaded information and communicated via email with practitioner.^18^ Of the trials that had included any measures of the consultation, five reported a difference in at least one of the included measures suggesting that the intervention may have had an effect on the consultation. (Table 4)

**Table 4:** Consultation measures to demonstrate evidence of impact of interventions on the consultation

Study: 1st author	Measures of the consultation	Reported differences in these measures between the intervention and control groups
Christian^31^	None reported	
Deakin^19^	No direct measure of the consultation but included patient‐completed questionnaires about treatment satisfaction, diabetes empowerment scores and diabetes knowledge scores	Significant differences in treatment satisfaction, diabetes empowerment and diabetes knowledge between groups
Kinmonth^23^	No direct measure of the consultation but included patient‐completed questionnaires with ratings of communication, satisfaction with treatment, style of care and knowledge of perceived control of diabetes	Intervention group report significantly higher communication scores with GPs, treatment satisfaction score and patient self‐reported knowledge score. No other measures demonstrated significant change between groups
Piatt^13^	No reported measures of consultation but included self report questionnaires on diabetes empowerment scale and diabetes knowledge test score	No statistically significant differences between groups in empowerment scale and diabetes knowledge test scores
Pill^21^	Direct measure of consultation through audio‐recordings. Also included qualitative feedback and questionnaire about understanding and implementation of intervention into practice from practitioners	Analysis of audio‐recording suggested more topic discussed with patients in intervention than control group. Patient participation in relation to “affirming health behaviours and initiating discussions of change” were significantly better in intervention than control group
Ralston^18^	No direct measure of consultations but reviewed patient activation by assessing number of clinic appointments, number of emails exchanged with practitioner and number of times that participants looked at e‐records and uploaded their blood glucose levels onto the web	Although no difference overall in the way health‐care services were used between intervention and control group, there is some evidence of effect on consultation from self‐report data. Care manager suggested an average of 4 hours per week updating care plans and communicating with patients over the web in the intervention group, and 76% of intervention group accessed their medical records
De fine Olivarius^22^	No direct measure of consultation but did include questionnaire on practitioners’ perceptions of participation, motivation and attitudes of their patients. Data also collected on differences in the way health‐care services were used by patients in terms of diabetes annual and three‐monthly review attendance and total number of consultations	Significant differences in practitioners’ perceptions of patient participation and motivation between groups. There were also significant differences in the attendance and number of consultations by patients between groups

### 3.3 Quantitative data synthesis

Overall, interventions to alter patient‐practitioner interactions were not significantly associated with difference in HbA_1c_ levels (weighted mean difference, −0.22%; 95% CI: [−0.56, 0.12]; *P*=.21; *I* ^2^=80%), SBP (weighted mean difference, −1.87 mm Hg; 95% CI: [−4.87, 1.13]; *P*=.22; *I* ^2^=49%), DBP (weighted mean difference, 0.03 mm Hg; 95% CI: [−1.44, 1.50] *P*=.96; *I* ^2^=39%), TC (weighted mean difference, −0.03 mmol/L; 95% CI: [−0.24,0.18] *P*=.81; *I* ^2^=53%), LDL‐C (weighted mean difference, 0.20 mmol/L; 95% CI: [−0.20, 0.60] *P*=.33 *I* ^2^=86%) and HDL‐C (weighted mean difference, 0.03 mmol/L; 95% CI: [−0.04, 0.09] *P*=.41 *I* ^2^=0%) when compared with usual care.

**Figure 3:**
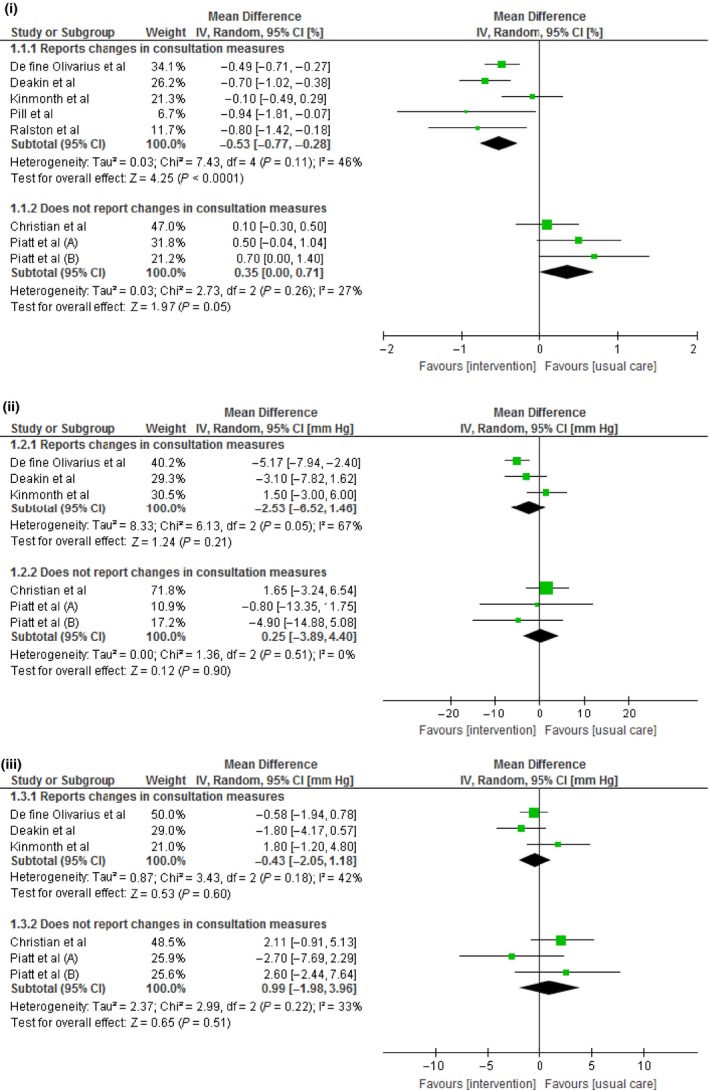
Forest plots of the effect of interventions to alter consultations between practitioners and type 2 diabetes patients, showing differences in outcomes of trials with and without demonstrable impact on the consultation. (i) Effect of interventions to alter consultations on HbA_1c_ levels (ii) Effect of interventions to alter consultations on systolic blood pressure. (iii) Effect of interventions to alter consultations on diastolic blood pressure

In subgroup analyses to explore heterogeneity, we found that interventions with measurable impact on the consultation were associated with reduced HbA_1c_ levels (weighted mean difference, −0.53%; 95% CI: [−0.77, −0.28]; *P*<.0001; *I* ^2^=46%) and a trend towards reduced blood pressure levels which did not reach statistical significance: SBP (weighted mean difference, −2.53 mm Hg; 95% CI: [−6.52, 1.46]; *P*<.21; *I* ^2^=67%) and DBP (weighted mean difference, −0.43 mm Hg; 95% CI: [−2.05, 1.18]; *P*<.18; *I* ^2^=42%). (Figure 3). Heterogeneity was reduced in this subgroup from high to moderate levels. None of the other remaining specified subgroup analyses reduced heterogeneity according to the *I* ^2^ statistic. There were too few trials reporting lipid levels for meaningful subgroup analysis of these outcomes. Finally, we included one study that was not set in primary care although comparable to this setting; removal of this study from the analysis made no difference to our findings.

### 3.4 Publication bias

Funnel plots suggested a low likelihood of publication bias for outcomes apart from lipid levels where there were too few studies to assess.

## 4 DISCUSSION

### 4.1 Summary

To our knowledge, this is the first meta‐analysis of RCTs examining the effect on CVD risk factors of interventions to alter consultations between primary care practitioners and patients with type 2 diabetes. We identified seven studies including 2277 participants with type 2 diabetes. Overall, interventions to alter consultations were not associated with improvements in CVD risk factors. However, trials were heterogeneous and subgroup analyses highlight the potential for important benefit on glycaemic control of interventions that demonstrated impact on the consultation (reductions in HbA_1c_ levels of −0.53% (5.7 mmol/mol)). There is a need for further research that includes consistent use of valid and reliable measures to demonstrate effect on consultations, alongside better development and incorporation of theory to elucidate mechanisms.

### 4.2 Strengths and limitations

Main strengths are that we conducted a systematic search of the literature that included no language or publication restrictions. We searched the grey literature, included studies identified in previous reviews and conducted hand searching of the literature to capture all relevant trials. It is possible that further trials have been conducted since our literature search. However, our electronic search updates since this time do not suggest additional published trials that would meet the inclusion criteria of our review. Limitations include the fact that the term “consultation” is a broad and multifaceted concept with various naming conventions and theoretical models.^3^, ^12^, ^24^, ^25^, ^26^ It is possible, therefore, that our search strategy may not have identified all relevant trials. As our search identified over 6000 titles, we did not extend it to include social sciences and non‐English language databases. However, the symmetrical funnel plot does not suggest that we have overlooked numerous negative trials. Only one of five authors approached responded to our requests for further information. We therefore extracted data and interpreted these without reference to original authors which could have introduced uncertainty in the meta‐analysis.^14^ However, there is recent evidence in favour of imputation methods within systemic reviews as preferable to omitting trials which could have led to a greater level of inaccuracy.^17^ We conducted multiple exploratory subgroup analyses and cannot exclude the role of chance as a plausible explanation for our findings. In our subgroup analysis, we pooled together studies that were likely to have altered the consultation based on the inclusion and reporting of consultation measures. However, it is plausible that some of the studies that did not include any measures of the consultation may still have had an effect on the consultation. Finally, there are many different ways to alter the consultation and the interventions reflected this; consequently, between‐study heterogeneity was high which is an argument against meta‐analysis.

### 4.3 Comparison with existing literature

A previous review, conducted over a decade ago, suggested that altering the consultation may have a clinically important effect on HbA_1c_ levels and diabetes outcomes.^5^ However, this was a descriptive review with no meta‐analysis and it combined primary and secondary care settings. A more recent meta‐analysis of the effect on health outcomes of interventions to alter the consultation again reported a similar clinically important effect that favoured interventions.^9^ This was conducted across multiple health conditions with only three trials in the review that were related to type 2 diabetes. Our current meta‐analysis builds on our earlier work and these reviews by including additional and larger trials that are specific to type 2 diabetes and primary care.^8^

It has been 10 years since our last review, and some progress has been made; five additional trials have been published measuring health outcomes in type 2 diabetes, and some trials included measures of process. However, there is still work to be done; trials are not consistently including and reporting measures of the consultation to demonstrate that interventions actually alter consultations. While there are a multitude of recognized patient‐centred consultation measures within the literature, none of these feature in included trials.^25^ Further, while there is now a better understanding of complex behavioural interventions in the literature and improved frameworks to guide the design and planning of interventions since our last review, none of these frameworks seem to have been used within included trials.^27^, ^28^ This might be one explanation for the lack of overall effects observed in this and previous reviews.^28^, ^29^ A previous report suggested that the majority of systematic reviews involving complex behavioural intervention do not tend to report strong effects.^30^ Reasons for this are suggested to be that complex behavioural interventions tend to be poorly described and developed which is what we found in our review.^30^ More theoretical work is needed to better understand the aspects of the consultation that could be amenable to change, how these may be related to health outcomes of interest and how to effectively change these within an intervention.

Finally, our findings could help to explain the discrepancy in the literature between trial and observational evidence on the potential effect of consultations on health outcomes among people with type 2 diabetes.^3^, ^4^, ^5^, ^6^ Our findings suggest variable impact of interventions on the consultation as an explanation for previous inconsistency. However, there are additional plausible explanations that should be considered to provide a complete view. These could include consultation context (eg varying health literacy, language or mental capacity), reverse causality, potential confounding variables in trials compared to observational evidence and the limitation of the RCT design itself which imposes a structure on the patient‐practitioner interaction that is key to its control design.

## 5 CONCLUSIONS

More health care in the UK is being delivered in primary care. There are increasing calls from policymakers and health professionals for more enabling consultations that address both disease and illness experience in patients with type 2 diabetes. However, the step from concept to operation remains challenging. This review provides some evidence of the potential of the consultation in primary care to improve CVD risk factors. But there is a need for more innovation in diabetes care for the 21st century in relation to the consultation between patient and practitioner. In this review, we observed that interventions to alter consultations have now moved beyond the traditional face‐to‐face consultation between practitioner and patient and towards system redesign to enable patient participation, empowerment and the inclusion of the primary care multidisciplinary team. This in turn could augment the direct consultation between practitioner and patient with type 2 diabetes. Consequently, future well‐conducted RCTs that take context as well as relationship into account are needed. Trials should include better descriptions of interventions, theoretical frameworks to underpin the interventions and robust measures of consultations to confirm that key aspects of the consultation and their context are being successfully enhanced.

## COMPETING INTERESTS

We have read and understood the policy on declaration of interests and declare that we have no competing interests**.**

## AUTHORS' CONTRIBUTION

HDM contributed to the design of the review, extracted data, wrote the analysis plan, conducted the analysis, drafted and revised the manuscript. AJC extracted data, conducted analysis and revised the manuscript. SJG and ALK initiated the project, contributed to the design of the review and revised the paper. SJG is guarantor.
